# Effect of Al Element on Retained Austenite, Residual Compressive Stress, and Contact Fatigue Life of Carburized and Quenched 20MnCr5 Steel Gear

**DOI:** 10.3390/ma17235764

**Published:** 2024-11-25

**Authors:** Yong Chen, Li Luo, Yuquan Zhang, Xingyun Zhou, Deshan Zeng, Fucheng Yu

**Affiliations:** 1 State Key Laboratory of Featured Metal Materials and Life-Cycle Safety for Composite Structures, Guangxi University, Nanning 530004, China; 2 School of Mechanical Engineering, Guangxi University, Nanning 530004, China; luoli178050512@163.com (L.L.); cc_yfc@163.com (F.Y.); 3 Tianjin Key Laboratory of New Vehicle Power Transmission and Safety Technology, Hebei University of Technology, Tianjin 300130, China; zyq43996@163.com; 4 Ningbo UMD Automatic Transmission Co., Ltd., Ningbo 315800, China; zhouxingyun@geely.com (X.Z.); deshan.zeng@geely.com (D.Z.)

**Keywords:** Al element, carburizing and quenching, retained austenite, residual stress, contact fatigue life, FZG spur gear

## Abstract

To improve the contact fatigue life of gears, we studied the effect of adding a certain proportion of the Al element to a 20MnCr5 steel FZG spur gear under different heat treatment processes, characterizing the retained austenite and residual compressive stress on the tooth surface. The stability of the microstructure grain size on the gear surface under different heat treatment processes was studied, and the surface microstructure, phase structure, and composition of the gear were characterized using scanning electron microscopy (SEM) and X-ray diffraction (XRD). The changes in the retained austenite content and grain size on the gear surface at a microscale of 2–100 μm were investigated. In addition, this study revealed the effect of adding the Al element and the optimization of the carburizing and quenching process on the residual compressive stress on the gear surface at a depth range of 200–280 μm. The effect of higher residual compressive stress and fewer non-metallic inclusions on the gear surface on the stress intensity factor of fatigue crack propagation was considered, along with the effect of deeper hardened layers on the improvement in wear resistance. The experiments in this study significantly improved the contact fatigue life of 20MnCr5 steel gears.

## 1. Introduction

As a key component of the high-speed transmission system of mechanical equipment [1,2,3], gears are widely used in automotives, aerospace, rail transit, wind power, and other fields. With the progress and development of the machinery industry, a much higher contact fatigue life and better reliability of gears are required due to the meshing exciting force of transmission systems under high speeds and heavy loads [4,5,6]. According to failure statistics, gear fatigue failure accounts for 60% of the total failure probability [7]. These problems can be addressed by optimizing the carburizing hardening process and other strengthening methods [8,9,10,11]. The former is an important heat treatment process that can improve the geometric accuracy of the tooth surface [12,13,14].

Controlling the influence of retained austenite and residual stress on the high-cycle contact fatigue performance of low-carbon alloy steel gear surfaces has been an important issue in the fields of metal materials and gear heat treatment, both at home and abroad. At present, the main processes involve changing the alloying element proportions and optimizing the carburizing and quenching heat treatment process. In this study, an Al element is added to 20MnCr5 steel gear, and its optimization is investigated in terms of the quantity, form, stability, and distribution of retained austenite as a component phase that affects the mechanical properties of the gear. At the same time, the mechanism of the residual stress preventing the propagation of tooth contact fatigue cracks is explored. This study furthers the research conducted in this field and is of theoretical and practical significance.

In terms of the effect of alloying elements on the properties of steel, Das et al. [15] found that alloying elements such as Mn and Al can promote the mechanisms associated with hydrogen-induced embrittlement (HIE). Tang et al. [16] found that the addition of Al and Ti is recommended for improving the anticorrosive properties and thermal stability of recrystallized grains. Hao et al. [17] stated that the toughness of steel is mainly affected by the aspect ratio and the dimension perpendicular to the fracture surface of MnS inclusions. Wang et al. [18] found that the enrichment of Cr in the rust layer after Zr–Ti-RE deoxidation improved the compactness and contributed to a reduction in the localized corrosion rate and long-term corrosion rate. Babenko et al. [19] found that a small amount of boron (≤0.004%) can improve the corrosion resistance of 08Kh18N10 stainless steel. Wang et al. [20] studied the solute elements in microalloying steel, concluding that they affect the solid solubility of microalloying elements and thus affect second-phase precipitation in steels.

In order to investigate the effects of heat treatment process optimization, Ramesh et al. [21] found that the presence of surface-retained austenite did not significantly improve the fatigue performance of the power transmission shaft, while martensite significantly improved the fatigue performance from 12,000 to 35,000 cycles under a torque load of ±3100 N·m. Miao et al. [22] heat-treated 20MnCrS5 low-carbon steel under standard and optimized conditions, in which the combined effects of reduced area, increased compressive residual stress, and reduced retained austenite resulted in a strengthening effect. Ren et al. [23] found that the accumulation of fatigue damage was influenced by the volume of retained austenite, and different fatigue damage accumulations were observed under RCF and TRBF conditions, highlighting its complex effects under different load conditions. Li et al. [24] studied retained austenite and its stability on 60Si2Mn steel after quenching and fractionation. Relatively stable retained austenite with a volume fraction of 13.7% and a hardness of 41HRC was obtained. Zhu et al. [25] studied the effect of the mechanical stability of retained austenite in the carburized layer on wear properties. The wear mechanism of retained austenite in the sliding friction process was explained, which provides a basis for the reasonable control of retained austenite in production. He et al. [26] studied the effect of Nb and B microalloying on the wear and corrosion resistance of 20MnCr5 steel after carburizing at 950 °C. The combination of Nb and B microalloying reduces the amount of retained austenite, thereby further improving the wear resistance. Kumar et al. [27] conducted an experimental study on the influence of gear preheating on residual compressive stress control and quenching. The single-frequency mode, preheat quenching treatment, and compressive residual stress field of spur gears during spin hardening were analyzed. Abedrabbo et al. [28] analyzed surface integrity in terms of texture, residual stress, microstructure, and microhardness and observed the fracture surface through torsional fatigue tests to determine the crack initiation location. To determine the influence of grinding heat on surface residual stress, Lei et al. [29] established a mathematical model of 12Cr2Ni4A alloy steel ultrasonic-vibration-assisted ELID grinding (UVAEG) for hardened surface residual stress. Izowski et al. [30] conducted a computational finite element analysis of Pyrowear 53 steel through low-pressure carburizing and oil–gas quenching and studied the effects of process parameters on the final phase composition and hardness of the material. In order to effectively control the deformation of carburizing the surface of gears, Liu et al. [31] established a calculation model of the gear-hardening method using the finite element method. Due to the influence of the carbon content, the phase transition between the gear surface and the center was inconsistent, which was the key factor affecting the deformation. Li et al. [32] measured the phase-change expansion, phase-change plasticity, and thermal physical property parameters of different structures of two kinds of automotive transmission steels: 20CrMnTiH and 20MnCr5. The phase transformation properties of the two materials after carburizing and quenching and their effects on deformation and residual stress were verified through experiments and numerical simulation. Zhang et al. [33] used standard spur gear specimens made of 20MnCr5 alloy steel for experiments. The results of the analysis showed that the three-parameter Weibu11 distribution of the contact fatigue life of a 20MnCr5 carburized gear can best fit the given stress and has good accuracy and adaptability. Calabokis et al. [34] proposed a method for the comprehensive contact fatigue evaluation of 18CrNiMo7-6 and 20Mncr5 alloy steel gears on an FZG test bench. The results of a SEM/EDS analysis showed that the occurrence of plastic deformation should be considered as a wear mechanism in the microstress analysis equation.

However, at present, the stability of the microstructure grain size on the surface of a 20MnCr5 steel gear with 0.03% Al under different heat treatment processes, and the mechanism of controlling the effect of retained austenite content and residual compressive stress of the gear surface on the high-cycle contact fatigue life of the gear, are still unclear. Through the study of the control of the volume, size, shape, and distribution of the metallographic retained austenite on the gear surface by adding Al element to 20MnCr5 steel and using a higher carburizing temperature, shorter carbon potential diffusion time, and 200 °C low tempering, this study investigates the change mechanism of the hardness gradient of the gear surface and residual compressive stress at meshing wear depth, and also investigates the effect of retained austenite content, residual compressive stress, and gear surface hardness on the high-cycle contact fatigue life of the gear.

## 2. Materials and Methods

### 2.1. Gear Parameters

**Figure 1:**
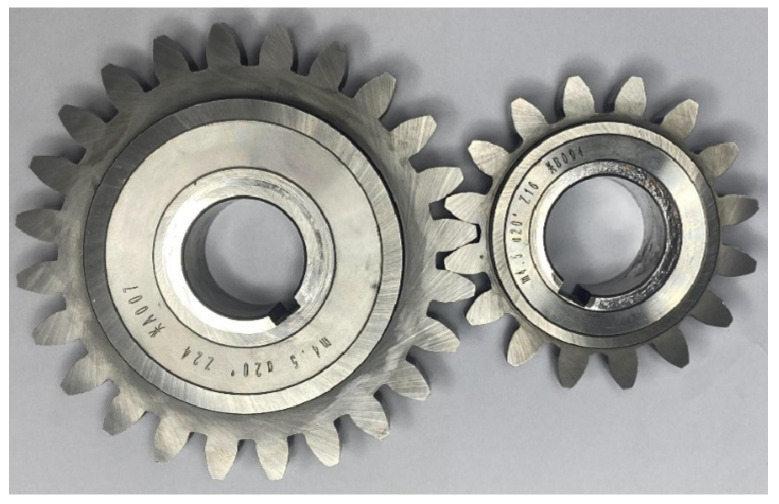
FZG-C gear.

This paper takes the FZG-C gear used in the FZG friction and wear testing machine as the research object; the gear is shown in Figure 1. The test gear is designed and manufactured strictly in accordance with the parameters of type C. The big gear has 24 teeth, the small gear has 16 teeth, and the pressure angle is 20°. The parameters of the gear are shown in Table 1.

### 2.2. Materials

The 20MnCr5 steel gear commonly used in automobile transmission systems was selected. The chemical element content of the 20MnCr5 steel gear is shown in Table 2. The gear studied in this paper included the addition of 0.03% Al element.

### 2.3. Gear Carburizing and Quenching Process

**Figure 2:**
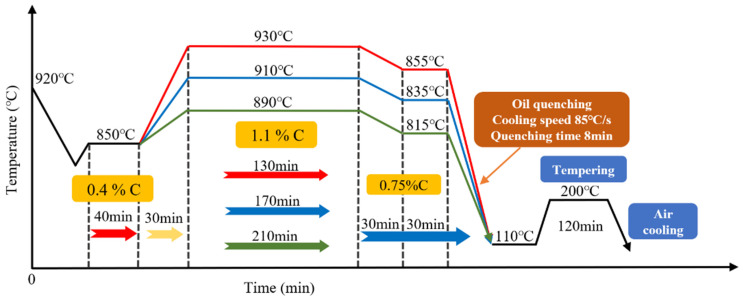
Carburizing–quenching process curve.

Chongqing Silver Shark Tools Co., Ltd. (Chongqing, China) carried out carburizing and quenching heat treatment on the experimental gear. The process curve for carburizing and quenching is shown in Figure 2. The gear was heated for 30 min to reach the carburizing temperature, and then the diffusion time at carburizing temperatures of 890 °C, 910 °C, and 930 °C and 1.1% carbon potential was 210 min, 170 min, and 130 min, respectively. The temperature was then lowered by 75 °C and the gear held at 0.75% carbon potential for 30 min. An RX atmosphere was used to obtain the carburizing atmosphere. The carbon potential was measured with an oxygen probe. The quenching oil temperature was 80 °C, the quenching cooling rate was 85 °C/s, and the quenching time was 8 min. The speed of the quenching agitator was 750 rpm and it was rapidly cooled to 110 °C in KR488 quenching oil. Finally, 200 °C low-temperature tempering for 120 min and air cooling were carried out.

## 3. Mechanism of Carburizing Phase Transition

### 3.1. Carbon Concentration

The process of carbon diffusion is often explained by Fick’s second law. Considering the diffusion rate and carbon concentration gradient of carbon in steel, the diffusion coefficient and transfer coefficient of carbon are determined:

(1) $$\frac{\partial C}{\partial t}-\frac{\partial }{\partial x_{i}}\left(D_{c}\frac{\partial C}{\partial x_{i}}\right)=0$$

where C is the carbon content of gear steel, t is the set carburizing time, $x_{i}$ is the position of carburizing, and $D_{c}$ is the carbon diffusion coefficient.

The carbon diffusion coefficient changes with the change in the carbon concentration, and the material alloy composition and carburizing temperature also affect the value of the carbon diffusion coefficient. The action equation is as follows:

(2) $$D_{c}=0.47p\cdot \exp\left(-1.6C\right)\cdot exp(-\frac{37000-6600C}{R_{c}T})$$

where $R_{c}$ is the gas-phase constant with a value of 1.986 cal/mol/K and p is the influence factor of the alloying elements.

The value of the alloying element influence factor is related to the type and content of the alloying element in the material. The calculation formula is as follows:

(3) $$p=1+\left(0.15+0.033Si\right)Si-0.0365Mn-\left(0.13-5.5^{e-3}Cr\right)Cr\;+\left(0.03-0.03365Ni\right)Ni-\left(0.025-0.01Mo\right)Mo-\left(0.03-0.02Al\right)Al\;-(0.016+1.4^{e-3}Cu)Cu-(0.22-0.01V)V$$

where Si, Mn, Cr, etc., are the mass fraction of each element in the steel gear.

It is necessary to determine the carbon concentration of the material before carburizing starts, and the carbon concentration is considered to be evenly distributed. The initial conditions are as follows:

(4) $$C{|}_{t=0}=C_{0}$$

where $C_{0}$ is the initial carbon concentration in the steel gear and the value is a constant.

The carbon atoms of the external carbon potential are constantly diffused to the inside through the gear surface, but due to the limitation of carburizing time and diffusion time, the depth of the carburizing layer is often a definite range, so when the distance from the surface reaches a certain depth or deeper, the carbon content at this position is still the original carbon content of the steel gear, and the internal boundary condition is as follows:

(5) $$C{|}_{x=x_{Max}}=C_{0}$$

where $x_{Max}$ is greater than the actual depth of the carburizing layer.

The ability of carbon atoms to transfer from the atmosphere to the gear is proportional to the difference in the carbon mass fraction between the external atmosphere and the gear surface, and the external boundary condition is

(6) $$-D\left(C\right)\frac{\partial C}{\partial x_{i}}=\beta_{C}(C-C_{w})$$

where $C_{w}$ is the carbon potential of the external atmosphere at $x_{i}$ and $\beta_{C}$ is the transfer coefficient of carbon atom from the atmosphere to the gear surface, which is affected by carburizing temperature, gas activity, and air pressure.

Due to the complex form of influence of the carbon transfer coefficient, only the influence of carburizing temperature is considered, and other values are taken as constants. The calculation formula is as follows:

(7) $$\beta_{C}=\beta_{0}exp(-\frac{E_{f}}{T_{a}R})$$

where $\beta_{0}$ is a constant related to material properties, and its value is 3.47 × 10^−3^ mm/s. $E_{f}$ is the activation energy of the reaction, and its value is 34 kJ/mol. $T_{a}$ is the carburizing temperature, and when $T_{a}$ is 1203 K, the transfer coefficient $\beta_{C}$ is 1.1587 × 10^−4^ mm/s. R is the molar gas constant and takes the value of 8.314 J/(mol·K).

### 3.2. Dynamic of Phase Transition

During the diffusion phase transition, the new phase is formed by the long-distance diffusion of atoms, and the transformation products are mainly bainite and pearlite. Considering the effects of carbon content, stress, and temperature, the Inoue model was adopted, and the volume fraction of the diffusive phase transition is expressed as follows:

(8) $$\xi_{B/P}=1-exp(-\int_{0}^{t}f_{1}(T)f_{2}(\sigma_{ij})f_{3}(C){\left(t-\tau \right)}^{3}d\tau )$$

where $\xi_{B/P}$ is the volume fraction of bainite and pearlite formation. $f_{1}(T)$, $f_{2}(\sigma_{ij})$, and $f_{3}(C)$ are functions of temperature T, stress $\sigma_{ij}$, and carbon content C, respectively.

Most phase transition processes form new phases through lattice shear and rotation, and the transformation products are mainly martensitic structures. In this study, the Inoue model is used to calculate the volume fraction of non-diffused phase transition:

(9) $$\xi_{M}=1-exp(\delta_{1}T+\delta_{2}(C-C_{0})+\delta_{3}\sigma_{m}+\delta_{4}\sigma_{e}+\delta_{5}))$$

where $\xi_{M}$ is the volume fraction of martensite formation; $\sigma_{m}$ is the average stress; $\sigma_{e}$ is the equivalent stress; and $\delta_{1}$, $\delta_{2}$, $\delta_{3}$, $\delta_{4}$, and $\delta_{5}$ are the test coefficients affected by temperature, carbon content, average stress, and equivalent stress, respectively.

### 3.3. Hardening Rule

The hardness value is estimated based on the superposition of each unit by the linear mixing principle. The hardness calculation of the gear steel after carburizing and quenching is as follows:

(10) $$H_{v}=\sum_{N=1}^{N_{P}}\gamma_{N}\xi_{N}+\sum_{K=1}^{K_{M}}\eta_{K}C_{K}$$

where $\xi_{N}$ is the volume fraction of different iron and carbon phases; $\gamma_{N}$ is the hardness of different iron and carbon phases; $\eta_{K}$ is the alloy component; and $C_{K}$ is the weight coefficient corresponding to the alloy composition.

The hardness calculation model of each iron phase structure is as follows:

Pearlite and cementite

(11) $$\gamma_{F-P}=42+223C+53Si+30Mn+12.6Ni+7Cr+19Mo\;+(10-19Si+4Ni+8Cr+130V)logV_{F-P}$$

Bainite

(12) $$\gamma_{B}=-323+185C+330Si+153Mn+65Ni+144Cr+191Mo+(89\;+53C-55Si-22Mn-10Ni-20Cr-33Mo)logV_{B}$$

Martensite

(13) $$\gamma_{M}=127+949C+27Si+11Mn+8Ni+16Cr+21logV_{M}$$

where $\gamma_{F-P}$, $\gamma_{B}$, and $\gamma_{M}$ are the hardness values of ferrite and pearlite, bainite, and martensite, respectively. $V_{F-P}$, $V_{B}$, and $V_{M}$ are the cooling rates of ferrite and pearlite, bainite, and martensite, respectively.

When C ≥ 0.5%, the hardness formula for martensite shown above is not applicable. In this case, the following formula is used:

(14) $$\gamma_{M}=\left(\frac{4+213C^{3}}{0.1+3.2C^{3}}-2C+1.5\right)\times 1.06$$

After carburizing and quenching, most of the austenite structure on the gear surface has completed the transformation to martensite, but some austenite still exists, and the existence of this retained austenite will also have a certain impact on the hardness of the gear. The calculation formula for retained austenite hardness is as follows:

(15) $$\gamma_{RA}=(\frac{\xi_{RA}}{1+0.2\xi_{RA}})\times 1.06$$

Here, $\gamma_{RA}$ is the hardness of the retained austenite and $\xi_{RA}$ is the volume fraction of the retained austenite.

## 4. Test and Verification

**Figure 3:**
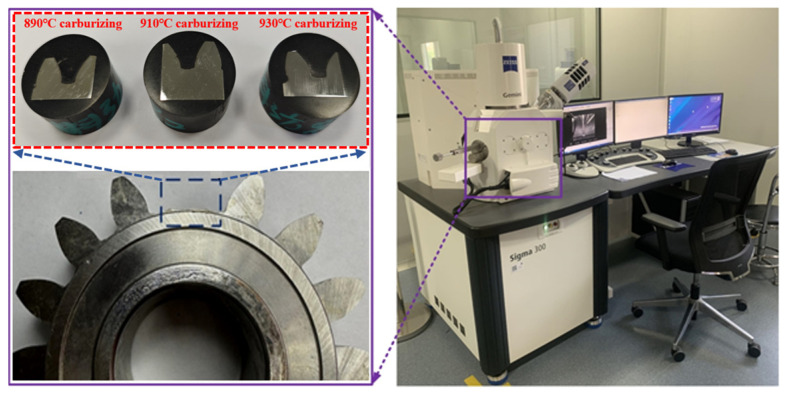
Microstructure test of carburized gear surface.

In order to test the carburizing diffusion, microstructure change, hardness change, martensite, and retained austenite volume of the 20MnCr5 steel FZG gear, the full meshing teeth of the gear were cut and inlaid. The surface of the gear sample was polished step by step with silicon carbide sandpaper to 1500 mesh, and then polished to a mirror surface with diamond paste (W2.5) and water. The sample was then ultrasonically cleaned with anhydrous ethanol solution, blow-dried, and acid-etched with 4% (volume fraction) nital. Finally, the microscopic structures such as retained austenite, martensite, and bainitic ferrite on the surface of the 20MnCr5 steel gear were characterized by scanning electron microscopy (SEM). The gear test sample is shown in Figure 3.

### 4.1. Carburizing Diffusion and Tissue Changes

**Figure 4:**
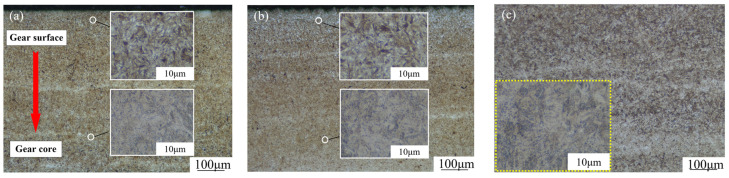

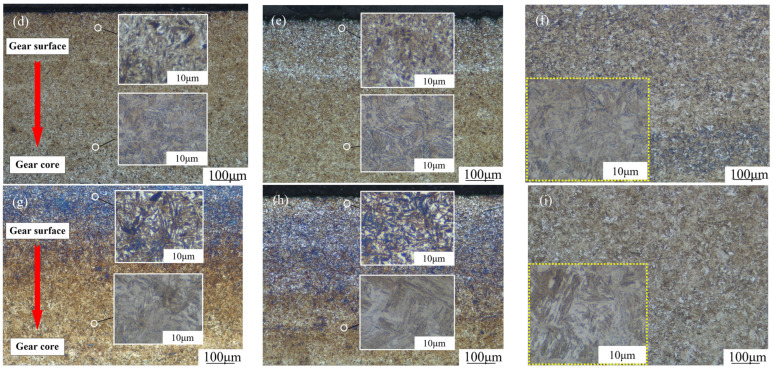
Microscopic morphology of tooth tip, reference circle, and core; 890 °C carburization (**a**–**c**); 910 °C carburization (**d**–**f**); 930 °C carburization (**g**–**i**); (**a**,**d**,**g**) surface layer at the tip circle; (**b**,**e**,**h**) surface layer at the reference circle; and (**c**,**f**,**i**) core.

**Figure 5:**
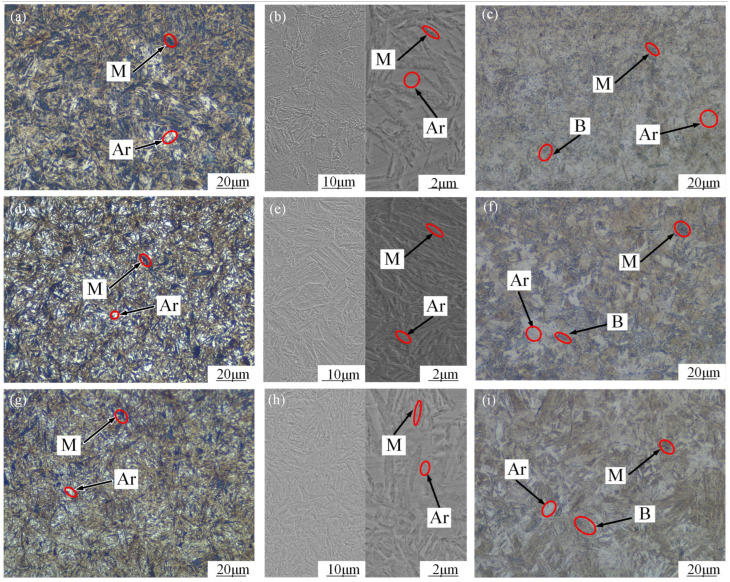
Microscopic morphology of gear surface and core; 890 °C carburization (**a**–**c**); 910 °C carburization (**d**–**f**); 930 °C carburization (**g**–**i**); (**a**,**b**,**d**,**e**,**g**,**h**) surface layer; and (**c**,**f**,**i**) core.

The surface morphology of the gear after carburizing was observed using scanning electron microscopy (SEM). The microstructure distribution and surface microstructure of the 20MnCr5 steel gear with Al element after different carburizing and quenching processes are shown in Figure 4 and Figure 5. Figure 4a,d,g, Figure 4b,e,h, and Figure 4c,f,i show the locally enlarged microstructure of the tooth tip, reference circle, and core at the scale of 100 μm and 10 μm, respectively. It can be seen that the surface microstructure of the 20MnCr5 steel gear after the optimized treatment is composed of a large amount of fine martensite, a uniform distribution of fine carbide, and a small amount of retained austenite. The tissue gradually changes from high-carbon martensite to low-carbon martensite from the tooth tip to tooth core. At the same time, there are obvious traces of carburizing diffusion in the tooth tip and the reference circle, and the core structure is more uniform.

As shown in Figure 4a,d,g, a higher carburizing temperature is conducive to the diffusion and plastic deformation of retained austenite. With the continuous accumulation of dislocation, which promotes the generation of nucleation, the transformation of austenite to martensite is induced; thus, the plastic deformation zone of the gear surface (TRIP effect) is strengthened. After carburizing at 930 °C, carbon potential diffusion for 130 min, and tempering at 200 °C, finer acicular-like martensitic structures appeared on the surface of the tooth tip, and the retained austenite volume was reduced and distributed evenly. This indicates that the grain size of the retained austenite does not increase due to the shorter carbon potential diffusion time, so higher temperature carburization, a shorter carbon potential diffusion time, and low-temperature tempering result in the uniform distribution of smaller carbides on the tooth tip surface.

It can be seen from Figure 4b,e,h that at the gear reference circle, from the tooth surface to the core, the high-carbon martensite structure is still gradually transiting to low-carbon martensite. With an increase in the carburizing temperature, the carbon potential diffusion time and low-temperature tempering heat treatment continue to produce dislocation accumulation on the tooth surface, and part of the retained austenite continues to transform into acicular martensite structures. It can be seen from Figure 4h that after carburizing at 930 °C, carbon potential diffusion for 130 min, and tempering at 200 °C, the quantity of martensite on the gear reference circle surface is significantly higher and finer, and the quality of the retained austenite continues to decrease.

As can be seen from Figure 4c,f,i, the tooth core is composed of strip martensite and partial bainite distributed along the grain boundaries. The martensitic structure of the tooth core is similar under different treatment processes, and the size of the strip martensite in the core structure will affect its hardness and strength. Because the temperature change rate of the tooth core is much less than that of the tooth surface, and it is difficult for the carburizing carbon potential to reach the tooth core, the hardness of the tooth core is basically the same under different processes: 416 HV, 419 HV, and 418 HV when carburizing at 890 °C, 910 °C, and 930 °C, respectively.

It can be seen from Figure 5a,d,g that there is more retained austenite at the scale of 20 μm at 890 °C. Figure 5b,e,h show that at 890 °C, there is more retained austenite at the scales of 10 μm and 2 μm. At 910 °C and with a shorter carbon potential diffusion time, the retained austenite gradually decreases and the martensitic structure becomes finer. At 930 °C, the carbon potential on the gear surface is further diffused. With a shorter diffusion time, the retained austenite is further reduced, its size does not increase, and there are more martensitic tissues distributed in fine needles. The results show that carburization at 930 °C, carbon potential diffusion at 130 min, and tempering at 200 °C can effectively reduce the retained austenite volume and improve the distribution state. As can be seen from the microstructure of the tooth core at the scale of 20 μm in Figure 5c,f,i, the carbon potential did not spread to the gear core after carburization at 890 °C, 910 °C, and 930 °C, and the tooth core mainly showed martensite and bainite microstructures with good toughness.

Krbata and Suski et al. [35,36] found that the austenitic grain size of low-carbon steel increases with the increase in austenitizing temperature in the temperature range of 800–1200 °C. In this study, under the conditions of adding 0.03% Al element, a higher carburizing temperature, shorter carbon potential diffusion time, and low-temperature tempering, a finer surface structure, greater martensite volume, and less retained austenite are obtained, in which the grain size is not increased; the size, state, and distribution of carbide are significantly improved; and the metallography structure distribution is more uniform.

### 4.2. Hardness Gradient

**Figure 6:**
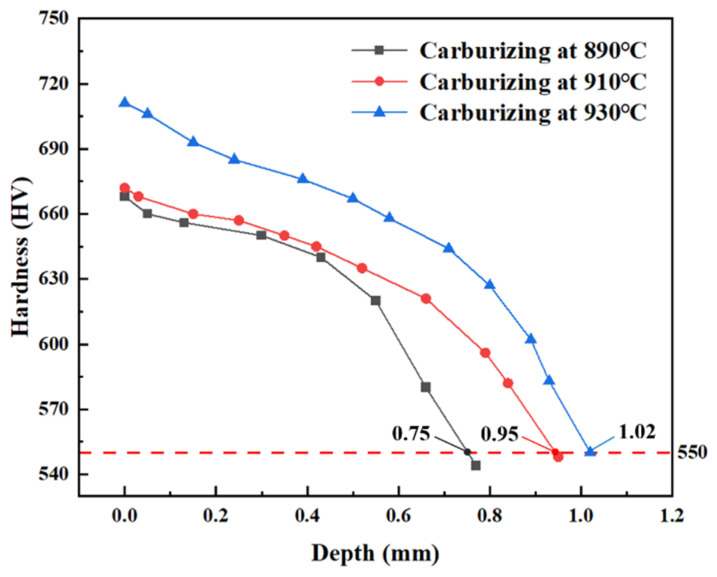
Hardness gradient of gear carburizing layer after carburizing and quenching.

The surface hardness at the gear division circle was analyzed and measured using a TUKON2500-6 microhardness tester (Laizhou Huayin test Instrument Co., Ltd., Yantai, Shandong, China) with a load of 1 kg for 10 s. Figure 6 shows the surface hardness gradient curve of the 20MnCr5 steel gear with Al element at the grading circle position under different heat treatment processes. It can be seen that the surface hardness at 890 °C, 910 °C, and 930 °C carburization is 668 HV, 672 HV, and 711 HV, respectively. When the effective hardness of the gear surface is ≥550 HV, it decreases with the increase in depth. This is due to a large amount of martensite formed by the high carbon content on the surface. From the surface to the tooth core, the martensite volume fraction and carbon content are reduced, resulting in high tooth surface hardness and low tooth core hardness. At the same depth of gear surface hardness (>550 HV), the hardness at 930 °C is generally higher than that at 910 °C and 890 °C. At 890 °C, 910 °C, and 930 °C, and when the carbon potential diffusion time is 210 min, 170 min, and 130 min at 200 °C for low-temperature tempering, the hardening depth is 0.75 mm, 0.95 mm, and 1.02 mm, respectively. Hence, a higher carburizing temperature, shorter carbon potential diffusion time, and low-temperature tempering can effectively increase the depth of the hardened layer of the gear meshing surface, thereby improving the wear resistance of the gear.

### 4.3. Retained Austenite Volume

In this study, the retained austenite on the gear surface was measured by X-ray diffraction (XRD) [37]. According to the principle of X-ray diffraction, the cumulative intensity of the X-ray diffraction lines of the material phase on the gear surface increases with the increase in the relative content of the phase in the sample. The cumulative intensity of the selected α- and γ-phase diffraction lines is substituted into Equation (16), and the volume fraction of the γ phase in the gear sample is calculated:

(16) $$V_{\gamma }=\frac{1-V_{c}}{1+G\frac{I_{{\alpha (hkl)}_{i}}}{I_{{\gamma (hkl)}_{j}}}}$$

where $V_{\gamma }$ is the volume fraction of the γ phase; $V_{c}$ is the volume fraction of the total carbide phase; $I_{{\alpha (hk1)}_{i}}$ is the cumulative intensity of the diffraction lines of the α-phase ${\left(hkl\right)}_{i}$ crystal plane; $I_{{\gamma (hkl)}_{j}}$ is the cumulative intensity of the diffraction lines of the γ phase ${\left(hkl\right)}_{j}$; and G is the ratio of the intensity-related factors of the γ-phase ${\left(hkl\right)}_{j}$ crystal face to the α-phase ${\left(hkl\right)}_{i}$ crystal face, which is $G_{{\alpha (hkl)}_{i}}^{{\gamma (hkl)}_{j}}$.

(17) $$G=\frac{V_{\alpha }}{V_{\gamma }}\cdot \frac{P_{\gamma {\left(hkl\right)}_{j}}}{P_{{\alpha \left(hkl\right)}_{i}}}\cdot \frac{{\left(L•P\right)}_{\gamma {\left(hkl\right)}_{j}}}{{\left(L•P\right)}_{{\alpha \left(hkl\right)}_{i}}}•\frac{e_{\gamma }^{-2M}}{e_{\alpha }^{-2M}}•\frac{{\left|F\right|}_{\gamma {\left(hkl\right)}_{j}}^{2}}{{\left|F\right|}_{\alpha {\left(hkl\right)}_{i}}^{2}}$$

where $\left(L•P\right)$ is the Lorentz polarization factor; P is the multiplicity factor of the crystal face; $e^{-2M}$ is the Debye–Vallow temperature factor; ${\left|F\right|}^{2}$ is the structure factor; and V is the cell volume.

In the process, the retained austenite produces plastic deformation. However, due to the continuous accumulation of dislocation, nucleation occurs, which induces the transformation of retained austenite into martensite, thus strengthening the plastic deformation zone. At the same time, martensitic transformation also occurs in the retained austenite after the gear surface wears down, forming a wear hardness gradient, and then it improves the wear resistance of the gear surface. In addition, in the process of transformation, the compressive stress inhibits crack nucleation and propagation due to the expansion of the structure, and improves the anti-crack propagation ability of the gear surface.

**Figure 7:**
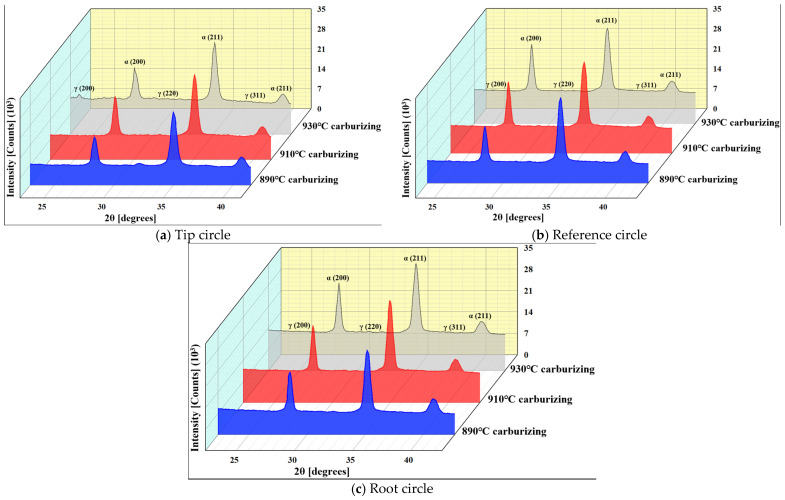
X-ray diffraction patterns of 20MnCr5 steel gear sample at 890 °C, 910 °C, and 930 °C carburizing temperature.

The X-ray diffraction patterns at 890 °C, 910 °C, and 930 °C of carburization are shown in Figure 7. The martensite is at the α(200) and α(211) crystal plane diffraction lines, and the retained austenite is at the γ(200), γ(220), and γ(311) crystal plane diffraction lines. The five diffraction lines are scanned step by step and the corresponding diffraction angle 2θ is determined. Due to the distance, the temperature of the tip circle drops rapidly during the quenching process, promoting the formation of more martensite, and the retained austenite at the tip circle is generally lower than at the reference circle and the root circle. Compared with carburization at 890 °C and 910 °C, the retained austenite of the tip circle, reference circle, and root circle at 930 °C, 130 min carbon potential diffusion, and 200 °C tempering is generally lower. This shows that the carbon potential diffusion and phase transition temperature occur at the tip circle, and the increased austenite phase transition forms fine-grained martensite.

Calculated using Equations (16) and (17), the retained austenite volume is shown in Table 3. It can be seen that the volume of retained austenite on the gear surface decreases with the increase in the carburizing temperature, and the retained austenite volume gradually increases from the tip circle to the reference circle and then to the root circle. This is because the tooth tip reaches the carburizing temperature faster, and the carbon potential diffusion rate and quenching cooling rate are also faster, in which the retained austenite volume at 930 °C is less than 8.5%. This verifies the fact that carburizing at 930 °C, carbon potential diffusion for 130 min, and low-temperature tempering at 200 °C can promote significant transformation from austenite to martensite.

### 4.4. Residual Stress

The long-term residual stress during the meshing of the gear pair changes the contact state of the gear surface and influences the high-cycle contact fatigue life of the gear. In the experiment, the surface of the 20MnCr5 steel gear under different heat treatment processes was subjected to 0–480 μm electrochemical corrosion with saturated sodium chloride solution, and the residual stress of the gear corrosion point was tested with a six-axis mechanical arm residual stress analyzer.

**Figure 8:**
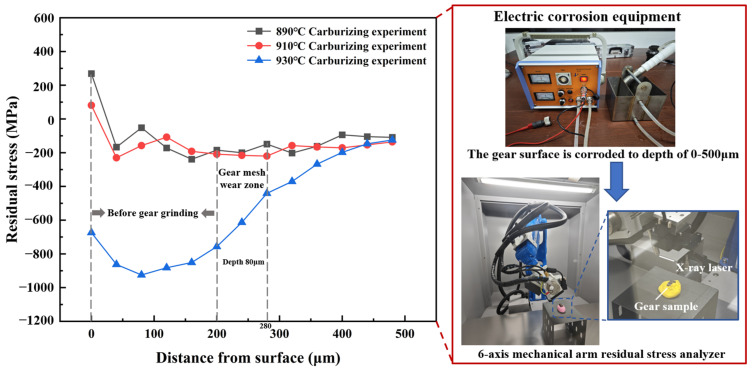
Test of electrochemical corrosion and residual stress on gear surface.

The results are shown in Figure 8. The residual stress increases first, and then decreases and tends to be stable with the distance from the tooth surface. Because the gear can be put into practical use when ground at a depth of 200 μm, the residual stress analysis is mainly carried out at the depth of 200~280 μm. It can be seen that with the increase in the carburizing temperature, the carbon potential diffusion time decreases and the residual stress increases overall in the area of actual meshing. After optimization at 930 °C, carbon potential diffusion for 130 min, and low-temperature tempering at 200 °C, the residual compressive stress at 200 μm can reach −757 MPa and at 280 μm can reach −441 MPa, increasing by 200~362%, which effectively hinders the propagation of contact fatigue cracks on the tooth surface and greatly reduces the propagation speed, so as to form a non-propagation crack. When the crack is generated at less than a certain depth of the reinforced layer, there will still be a certain residual compressive stress region at the crack tip. The high residual compressive stress can not only reduce the stress intensity factor controlling fatigue crack propagation, but also enhance the closing effect of fatigue cracks and increase the critical stress of fatigue crack opening, so that the contact fatigue life after optimization can be effectively improved.

## 5. Fatigue Test of Carburized and Quenched Gear

### 5.1. Experimental Equipment

**Figure 9:**
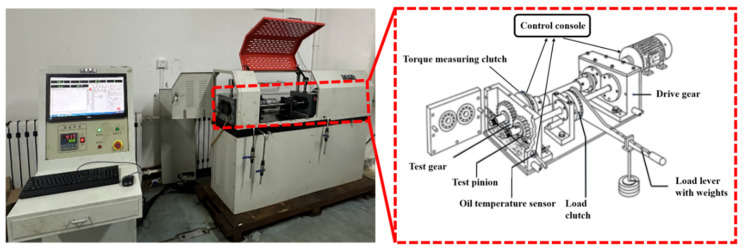
FZG friction and wear testing machine.

An FZG friction and wear test bench was used to carry out the gear contact fatigue experiment. The test bench and its components are shown in Figure 9. The testing machine is mainly composed of a test gear box, torque speed sensor, drive shaft, attendant gear box, temperature control system, and computer control panel. The gear box is lubricated with dipping oil. A heating device is installed at the bottom of the gear box to heat the oil, and the test bench adopts lever weight loading, which can load 12 different load stages.

### 5.2. Experimental Conditions

The experimental gear was tempered and ground after carburizing and quenching, and the experimental lubricating oil model was CECL-07-A-85. The experimental gears were carburized at 910 °C and 930 °C, with carbon potential diffusion at 170 min and 130 min and low-temperature tempering at 200 °C, respectively. The precision of the gear after grinding was 5. The contact fatigue strength limit of 20MnCr5 steel is 1400 MPa. When the experimental load is level 9, the contact stress borne by the driving pinion is 1395 MPa. When the contact stress of the tooth surface is close to or exceeds the contact fatigue strength limit of the steel, the tooth surface is prone to fatigue cracks [38]. Therefore, the contact fatigue life test of 910 °C and 930 °C carburized and quenched gears was carried out with a nine-stage load; the experimental groups are shown in Table 4.

## 6. Experimental Results and Discussion of Gear Fatigue Performance

### 6.1. Wear Resistance

The surface crack of the gear originates from the micro-convex or pitting pit. When the cumulative strain exceeds the critical value of the material, micro-crack initiation occurs. The sub-surface micro-cracks of the gears with medium and low paralympic content are connected together under shear stress and spread along the interface between the hardened layer and the substrate. When the crack extends to a certain depth, it breaks away from the Hertzian contact stress field and turns parallel to the surface, resulting in fatigue failure. When the retained austenite content is high, the original austenite grain boundary is greater, and the crack spreads through the grain boundary near the surface. After a certain depth, the crack energy weakens and results in intergranular expansion to a larger depth, so the residual compressive stress can reduce the stress intensity factor and enhance the closing effect of the fatigue crack to increase the critical stress of fatigue crack opening. The secondary crack induced by the main crack occurs due to the stress field and the grain boundary and spreads to the surface, resulting in fatigue spalling and pitting.

**Figure 10:**
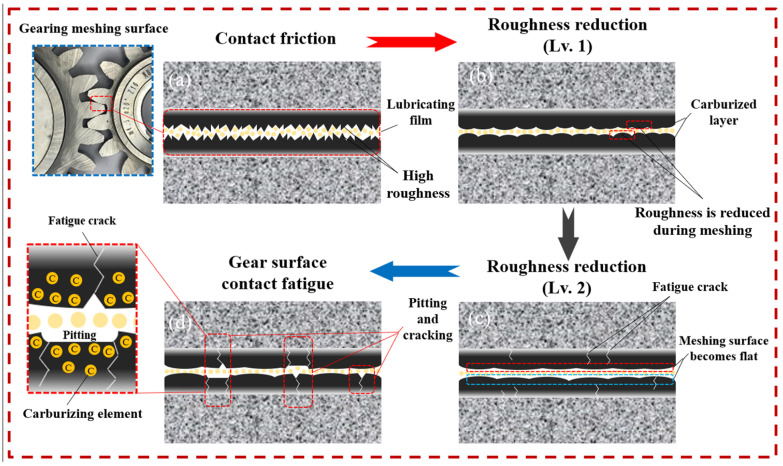
Failure mechanism of gear meshing pitting.

The failure mechanism is shown in Figure 10, and the initial state of the gear meshing surface microstructure is shown in Figure 10a. In the early stage, there are many small roughness peaks on the tooth surface, and the peak value is high. In the process of continuous meshing wear, the higher the surface hardness, the better the wear resistance and the better the surface integrity of the tooth surface morphology. As shown in Figure 10b, with the roughness peak gradually decreasing, the tooth surface becomes smoother, and the roughness value gradually decreases. Figure 10c shows that the meshing surface roughness is lower and micro-cracks appear. After continued meshing wear, as shown in Figure 10d, fatigue micro-cracks and pitting corrosion appear on the tooth surface, and then the damage is intensified until fatigue failure.

The test showed that the 20MnCr5 steel gear carburized at 910 °C exhibited single-tooth contact fatigue failure after 28 h of operation, but the gear carburized at 930 °C of quenching treatment still ran normally. In order to further verify and compare the tooth surface changes with different temperatures during the same running time, the roughness of all of the undamaged meshing tooth surfaces was tested after contact fatigue failure at 910 °C carburization.

**Figure 11:**
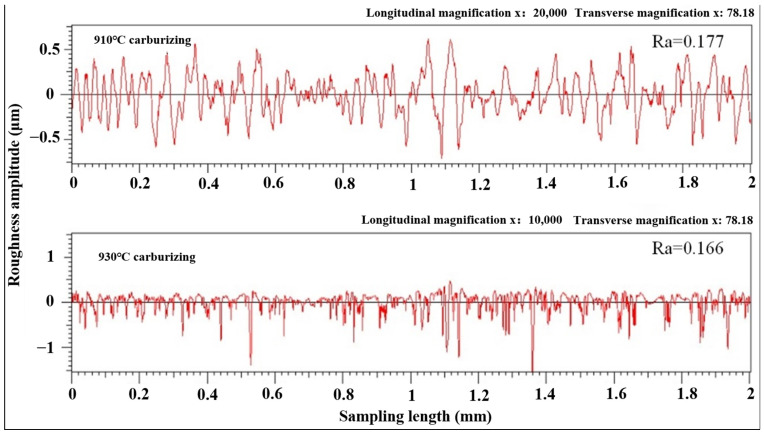
Roughness amplitude of tooth surface after experiment.

Figure 11 shows the roughness at 910 °C and 930 °C carburization after 28 h of operation. It can be seen that, compared with the gear before the experiment, the roughness Ra decreases significantly. Because of the tooth surface’s slip and roll, it continues to wear due to cyclic meshing force, and then the machining tool marks and convex peaks are smoothed, resulting in the reduction in the tooth surface roughness.

**Figure 12:**
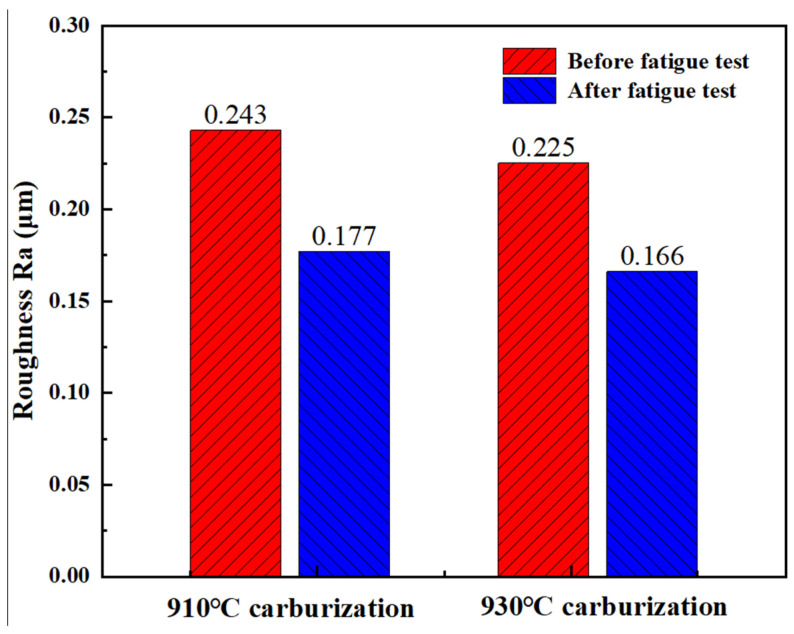
Variation in tooth surface roughness before and after fatigue test.

Figure 12 shows the roughness change during 28 h and before the test. By comparing Figure 11 and Figure 12, it can be seen that the peak roughness at 910 °C is higher and the roughness value declines rapidly after the experiment. After the carburizing treatment at 930 °C, it is lower and the decline is slower. This is because the higher the hardness of the tooth surface, the stronger the wear resistance, as the surface-processing tool marks are not easily worn down, and the peak roughness does not decline obviously. This is similar to the conclusion made by Aksoy and Xu et al. [39,40] that the increase in metal surface hardness improves the wear resistance.

### 6.2. Fatigue Life

**Figure 13:**
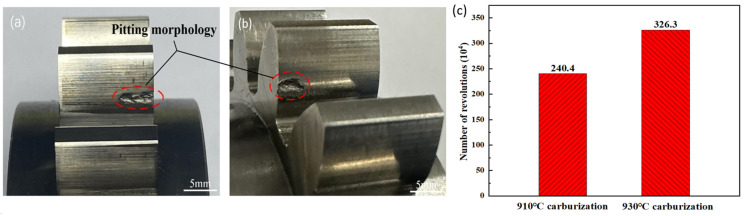
Macroscopic pitting morphology of gear after experiment: (**a**) carburizing and quenching at 910 °C, (**b**) carburizing and quenching at 930 °C, and (**c**) fatigue life.

Figure 13a,b show the macroscopic morphology of the fatigue pitting position of the carburized and quenched gear at 910 °C and 930 °C. It can be seen that the pitting positions of all gears have a trend of expanding from the lateral end face of the tooth surface to the inside of the gear and from near the root circle to the tip circle. The initial pitting positions are all located below the reference circle and near the root circle.

Figure 13c shows the contact fatigue life results of the 20MnCr5 steel gear after carburizing at 910 °C and 930 °C. It can be seen that the contact fatigue life of the gear after carburizing at 930 °C is significantly higher than that at 910 °C. At 910 °C, pitting failure occurred and the pitting area rate was approximately 11% after 2.404 million cycles, changing to 8% after 3.263 million cycles. Compared to carburization at 910 °C, the contact fatigue life at 930 °C increased by more than 35%. Therefore, the latter effectively reduces the retained austenite volume, forms a finer martensitic structure, and significantly increases the residual compressive stress, thereby improving the pitting and contact fatigue resistance.

### 6.3. Pitting Morphology Analysis

**Figure 14:**
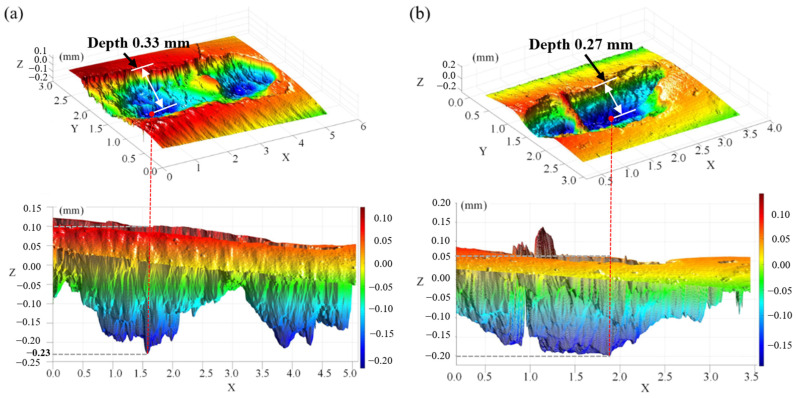
Three-dimensional morphology of gear pitting position: (**a**) 910 °C carburization and (**b**) 930 °C carburization.

The pitting depth and pitting peak of the gear after carburizing at 910 °C and 930 °C were analyzed using a MicroXAM-3D (Wuxi Auben precision Industry Co., Ltd., Wuxi, Jiangsu, China). Figure 14 shows the three-dimensional morphology of the pitting positions at 910 °C and 930 °C. There are multiple pits, varying in depth, each connected to each other to form larger pitting/spalling. After pitting failure, it can be seen that there are still many micro-cracks near the pit, and the cracks at the edge of the pitting position continue to expand to the tip ring and the middle of the tooth surface. Pitting and spalling occurred in some parts of the gear at 910 °C carburization: the two large pits were connected to each other, and the small pits were more distributed. Because the inclusions inside the material were relatively weak, the cracks spread along the inclusions after each micro-pitting, resulting in the middle of the pitting part of the tooth surface not falling off, but spalling along both sides, with a maximum pitting depth of 0.33 mm. At 930 °C, a small area split off with a maximum depth of 0.27 mm. A more uniform distribution, lower surface-retained austenite volume, and higher surface residual compressive stress result in better pitting resistance and significantly improve the contact fatigue performance of 20MnCr5 steel gears.

### 6.4. Microscopic Morphology Analysis

**Figure 15:**
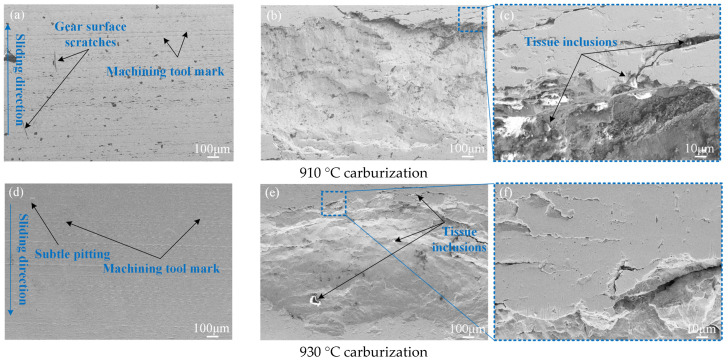
Meshing tooth surface and pitting microstructure of gear.

The detection and analysis of the pitting microstructure are shown in Figure 15. It can be seen that at both 910 °C and 930 °C carburization, a certain amount of tooth surface wear was produced. A small area showing the tool marks can be observed at 910 °C, indicating that greater wear has occurred. At 930 °C carburization, there is less wear than at 910 °C, although some small marks can still be observed. Due to the finer martensitic structure, strain-induced martensitic transformation (TRIP effect), and the stress-induced transformation of retained austenite to martensitic in front of the crack, the stress field at the crack front is effectively reduced, and the crack growth rate and pitting corrosion are delayed. As mentioned in Section 4.4 and Section 6.1, the overall strength, toughness, surface hardness, and fatigue life can be improved.

As can be seen from Figure 15a–c, there are many machining tool marks on the surface of the gear after carburizing at 910 °C and before meshing wear. With the action of continuous meshing wear and metal inclusions, pitting morphology appears gradually. Finally, fatigue cracks and large-area pitting/spalling are formed at the metal inclusions. Figure 15b–f show the microscopic morphology of the 20MnCr5 steel gear after pitting at 910 °C and 930 °C carburization. It can be seen that there are some spherical or multilateral particles on the surface, and the cracks continue to expand around them, which are judged to be non-metallic inclusions that will destroy the uniformity of the material where the stress concentration occurs. This is often the priority area for crack initiation, crack propagation, and pitting; therefore, inclusions affect the contact fatigue performance of the gears. This is consistent with the findings of Guo and Hashimoto et al. [41,42]: the smaller the grain size, the more difficult it is for fatigue cracks to appear.

The level of non-metallic inclusions was tested. The field of view of six groups of inclusions was obtained from the test, including Class A (sulfide) coarse 0.5 × 3 (4–12 μm) and fine 2.0 × 6 (2–4 μm); Class B (alumina) coarse 0 (9–15 μm) and fine 0.5 × 6 (2–9 μm); Class C (silicate) coarse 0 (5–12 μm) and fine 0 (2–5 μm); and Class D (spherical oxide) coarse 0.5 × 2 (8–13 μm) and fine 0.5 × 6 (3–8 μm). The inclusions were mainly fine sulfide, alumina, and spherical oxide. Lower inclusion levels also reduce the incidence of surface crack initiation and pitting corrosion, increasing the contact fatigue life of the gear.

## 7. Conclusions

In this study, we analyzed the surface contact fatigue strengthening mechanism of a 20MnCr5 steel FZG gear with trace Al element under different heat treatment processes and performed experimental verification on the high-cycle fatigue life of the gear, drawing the following conclusions:

1. Adding 0.03% Al element to 20MnCr5 steel gears leads to a finer surface structure, larger martensite volume, greatly reduced retained austenite content, and unchanged grain size of the retained austenite, and significantly improves the size, state, and distribution of carbides at a higher carburizing temperature, shorter carbon potential diffusion time, and low-temperature tempering process.
2. After the optimization of the carburizing and quenching process, the 20MnCr5 steel gear yields an effective hardened layer depth of 1.02 mm and a maximum surface hardness of 711 HV; the retained austenite content on the gear surface is less than 8.5%. A more uniform surface structure and higher residual compressive stress are obtained, and the maximum residual compressive stress in the meshing wear area is increased by 200~362%.
3. The higher hardness, lower retained austenite volume, and higher residual compressive stress on the gear surface effectively reduce the stress field of the crack front and enhance the closing effect of fatigue cracks. A lower inclusion level delays crack initiation and pitting. The number of gear contact fatigue pitting tests is increased from 2.404 million to 3.263 million and the gear contact fatigue life is increased by more than 35%.

## Figures and Tables

**Table 1:** FZG-C gear parameters.

Gear Parameter	Driving Gear	Driven Gear
Number of teeth	16	24
Coefficient of displacement	0.182	0.171
Tip circle diameter/mm	82.5	118.4
Module/mm	4.5	
Pressure angle/(°)	20	
Tooth width/mm	14	
Bore diameter/mm	30	

**Table 2:** Chemical composition of 20MnCr5 steel gear, wt%.

Steel	C	Si	Mn	P	S	Cr	Ni	Mo	Cu	Al
20MnCr5	0.18	0.09	1.27	0.012	0.02	1.22	0.04	0.01	0.01	0.03

**Table 3:** Retained austenite volume of carburization-hardened gear surface.

Retained Austenite Volume	Tip Circle (%)	Reference Circle (%)	Root Circle (%)
890 °C carburization	20.1	20.3	20.6
910 °C carburization	14.7	15.8	16.0
930 °C carburization	7.7	8.2	8.5

**Table 4:** Parameter settings for gear contact fatigue test.

Experimental Stage	Parameter Setting	Parameter Value
Run-in stage (2 h)	Rotational speed (rpm)	3000
	Torque (N·m)	135
	Oil temperature (°C)	90 ± 2
Experimental	Rotational speed (rpm)	1431
	Torque (N·m)	302
	Oil temperature (°C)	90 ± 2

## Data Availability

The original contributions presented in the study are included in the article, further inquiries can be directed to the corresponding author.
